# Luteoloside Acts as 3C Protease Inhibitor of Enterovirus 71 *In Vitro*

**DOI:** 10.1371/journal.pone.0148693

**Published:** 2016-02-12

**Authors:** Zeyu Cao, Yue Ding, Zhipeng Ke, Liang Cao, Na Li, Gang Ding, Zhenzhong Wang, Wei Xiao

**Affiliations:** State Key Laboratory of New-tech for Chinese Medicine Pharmaceutical Process, Jiangsu Kanion Pharmaceutical Co., Ltd., Lianyungang, Jiangsu, China; Centro de Biología Molecular Severo Ochoa (CSIC-UAM), SPAIN

## Abstract

Luteoloside is a member of the flavonoids family that exhibits several bioactivities including anti-microbial and anti-cancer activities. However, the antiviral activity of luteoloside against enterovirus 71 (EV71) and the potential mechanism(s) responsible for this effect remain unknown. In this study, the antiviral potency of luteoloside against EV71 and its inhibitory effects on 3C protease activity were evaluated. First, we investigated the cytotoxicity of luteoloside against rhabdomyosarcoma (RD) cells, which was the cell line selected for an *in vitro* infection model. In a subsequent antiviral assay, the cytopathic effect of EV71 was significantly and dose-dependently relieved by the administration of luteoloside (EC_50_ = 0.43 mM, selection index = 5.3). Using a plaque reduction assay, we administered luteoloside at various time points and found that the compound reduced EV71 viability in RD cells rather than increasing defensive mobilization or viral absorption. Moreover, biochemical studies focused on VP1 (a key structural protein of EV71) mRNA transcript and protein levels also revealed the inhibitory effects of luteoloside on the EV71 viral yield. Finally, we performed inhibition assays using luteoloside to evaluate its effect on recombinant 3C protease activity. Our results demonstrated that luteoloside blocked 3C protease enzymatic activity in a dose-dependent manner (IC_50_ = 0.36 mM) that was similar to the effect of rutin, which is a well-known C3 protease inhibitor. Collectively, the results from this study indicate that luteoloside can block 3C protease activity and subsequently inhibit EV71 production *in vitro*.

## Introduction

Luteoloside is a member of the flavonoids family and was extracted from several traditional Chinese herbs [1–4] and other plants [5,6]. Previous studies have reported the potential bioactivities of luteoloside, which include anti-microbial activity [3], the inhibition of melanogenesis [5], the suppression of proliferation [7], and anti-cancer activity [8]. Flavonoids exhibit broad-spectrum antiviral effects against influenza virus [2], human rhinovirus [9], poliovirus [10–13], coxsackievirus B4, echovirus 6, and enterovirus 71 (EV71) [11]. However, studies focused on the antiviral efficacy of luteoloside against EV71 are limited.

EV71 is a member of the *Picornaviridae* family and is the causative agent of hand, foot, and mouth disease (HFMD) in infants and young children. Without timely or effective treatments, EV71 induces severe nervous and respiratory system complications that lead to poor prognoses and high fatality rates [14]. For example, in 2012, a large EV71 outbreak in China caused more than 2 million clinical cases and 567 deaths [15]. However, there is currently no approved drug for the treatment of EV71 infection. Thus, identifying an effective EV71 candidate target for the prevention and treatment of infections is necessary.

The EV71 genome consists of approximately 7500 nucleotides and one open reading frame [16]. During translation, a single precursor polyprotein is synthetized and cleaved by proteases into four structural proteins (VP1–VP4) and seven functional proteins (2A–2C and 3A–3D) [17,18]. The majority of the viral epitopes are located on VP1, which is a key structural protein of EV71 [19–20]. Thus, VP1 is typically used as an indicator for the detection, identification, classification, and phylogenetic analysis of various EV71 genotypes [21–23]. Additionally, the 3C protease plays an important role in the life cycle of EV71. This protease cleaves the precursor polyprotein into individual proteins and interacts with several host factors that are critical to protein and nucleic acid synthesis [17, 24–27]. Moreover, the 3C protease induces apoptosis in host cells [28]. Taken together, these findings indicate that the 3C protease may serve as a novel target for the identification and development of anti-EV71 agents [29]. For example, rupintrivir, which is an inhibitor of the 3C protease, exhibits antiviral potency against many strains of EV [30,31]. Rutin is another well-known flavonoid compound and inhibits EV71 viability by blocking 3C protease activity [32]. However, the effects of luteoloside on 3C protease and subsequent viral propagation remain unknown and require further study.

Therefore, in the present study, we investigated the antiviral effects of luteoloside against EV71 and its use as a potential EV71 target. The data indicated that luteoloside is an inhibitor of 3C protease and suppresses EV71 production *in vitro*.

## Materials and Methods

### Chemicals

Luteoloside (C_21_H_20_O_11_; CAS No. 5357-11-5; MW 448.38; ≥99% pure) was purchased from the National Institutes for Food and Drug Control of China. The MTS cell proliferation assay kit was obtained from Promega Biotech Co., Ltd. (Beijing, China). The other reagents (AR grade) were obtained from Sinopharm Chemical Reagent Co., Ltd. (Shanghai, China), unless stated otherwise.

### Cells and virus

Rhabdomyosarcoma (RD) cells were chosen as the infection model [33, 34], purchased from the American Type Culture Collection and cultured in Dulbecco’s modified Eagle’s medium (DMEM) supplemented with 10% foetal bovine serum (FBS, Gibco) at 37°C in a humidified incubator with 5% CO_2_. A clinically isolated EV71 strain (GenBank accession No. HQ882182) was kindly supplied by Dr. Xilang Wang from the Institution of Microbiology and Epidemiology, Academy of Military Medical Sciences, People's Liberation Army of China (Beijing, China). EV71 was propagated in RD cells, and the titres were determined by measuring the 50% tissue culture infective dose (TCID_50_) per millilitre as previously described [35].

### Cytotoxicity assay

The dose of luteoloside required for 50% cell cytotoxicity (CC_50_) of RD cells was analysed as reported previously [36]. Briefly, RD cells (10^4^ cells·well^-1^) were seeded in 96-well plates and treated with luteoloside in a range of concentrations from 0.0625 to 4.0 mM (dissolved in DMEM medium containing 2.5% FBS) in triplicate. The cell viabilities were determined using MTS cell proliferation assay kits at 48 hours after the addition of the compound.

### Antiviral assay

For the antiviral assay, RD cells (10^4^ cells·well^-1^) were plated in 96-well plates with DMEM medium and then infected with EV71 at a multiplicity of infection (MOI) of 0.3 or 1.0 at 37°C for 2 h. After removing the virus, the infected cells were supplemented with luteoloside (dissolved in DMEM medium containing 2.5% FBS) at different times as indicated. The compound was diluted with medium and then used at various doses in triplicate. Cell viability was estimated using the MTS cell proliferation assay kit according to user manual at 48 hours post-infection (hpi). The concentration that elicited 50% of the maximal effect (EC_50_) and the selection index (SI) were calculated. For the RNA and protein extractions, the RD cells (1×10^6^ cells·well^-1^) were plated in 6-well plates and treated with 0.5 mM luteoloside at 37°C for 2 h. After removing the compound, the RD cells were inoculated with 0.3 MOI EV71 37°C for 2 h.

### Inhibition of different stages of viral production by luteoloside

To determine the viral production stage during which luteoloside exhibited its inhibitory effects, a time-of-addition experiment was performed. In Protocol 1, RD cells (10^4^ cells·well^-1^) were plated in 96-well plates with DMEM medium containing 0.5 mM luteoloside at 37°C for 2 h. After removing the compound, the RD cells were inoculated with 0.3 MOI EV71 at 37°C for 2 h. Then, the virus suspension was washed out, and DMEM medium containing 2.5% FBS was added. In Protocol 2, a mixture of EV71 (MOI = 0.3) and luteoloside (0.5 mM) was added to RD cell monolayers and incubated at 37°C for 2 h. After removing the medium, DMEM medium containing 2.5% FBS was added. In Protocol 3, after absorption of the EV71 virus (MOI = 0.3) at 37°C for 2 h, 0.5 mM luteoloside dissolved in DMEM medium containing 2.5% FBS was supplied to RD cells. In all of the protocols, cell viability was estimated using MTS cell proliferation assay kits according to user manual at 48 hpi.

### Time-course analysis of EV71 production

To ascertain the inhibitory effects of luteoloside on EV71 production, plaque assays were performed. Infected RD cells (1×10^6^ cells·well^-1^) seeded in 6-well plates were treated with or without 0.5 mM luteoloside. The intercellular and extracellular virions were collected by freeze-thawing at -80°C. The plaque reduction assays were performed using 2-fold dilutions in triplicate. Confluent monolayers of RD cells in 6-well plates were incubated with each sample in 0.1 mL for 2 h. After removing the samples, all wells were supplemented with DMEM medium containing 1% methyl cellulose and 2% FBS. The medium was removed after treatment at 37°C for 3 days. The cells were fixed with PBS containing 20% formaldehyde and 1% crystal violet for 1 h at room temperature. Then, the plaques were counted.

### Viral load detection

Total RNA was isolated from the RD cells using the TRIzol reagent (Invitrogen, USA), and cDNA was synthesized using random hexamers with a reverse transcription kit (TaKaRa, China) according to the user manuals. The cDNA was examined with an EV71 RNA Detection Kit (Shanghai ZJ Bio-Tech Co., Ltd) specific for the *VP1* gene. The positive fragments observed over a series of concentrations were used to create a standard curve.

### Western blot analysis of VP1

The cells were treated with RIPA lysis buffer (Beyotime Institute of Biotechnology, China). The lysates were centrifuged at 12000 g for 10 min at 4°C. The total protein concentrations were determined with a bicinchoninic acid protein assay kit (Beyotime Institute of Biotechnology, China). All protein samples were standardised to 40 mg and subjected to SDS-PAGE using 12.5% acrylamide resolving gel and transferred to a PVDF membrane (Millipore, USA). The membrane was blocked with 2% non-fat dried milk solution in Tris-buffered saline containing 0.1% Tween-20 for 2 h and then incubated overnight at 4°C with primary antibody against VP1 (Abcam, UK). Immune complexes were detected using HRP-conjugated rabbit anti-mouse IgG. The band was visualized with a solution containing 3 mM 3,3’-diaminobenzidine tetrahydrochloride (Sigma, USA) and 1 mM H_2_O_2_. The expression level of β-tubulin was utilized as an internal standard. The band intensity was analysed using Image Lab^TM^ Software.

### 3C protease activity assay

The activity of the 3C protease was analysed according the method detailed in a previous study with slight modifications [32]. Briefly, horseradish peroxidase type VI-A (HRP, Sigma, USA) containing the cleavage site of the 3C protease (at the Gln-Gly residues) was designed as the substrate. Recombinant EV71 3C protease (HQ882182) was supplied by Zoonbio Biotechnology Co., Ltd. The 3C protease (0.1 μM) and HRP (0.1 mg/L) were incubated for 2 h at 37°C in 96-well plates *in vitro*. The remaining activity was assessed using ABTS [2,2’-azino-di-(3-ethyl-benzthiazoline-6-sulphonic acid)] and H_2_O_2_ and monitored at A_405_. To determine the inhibitory effects, 2-fold dilutions of luteoloside were added to the 3C protease analysis system, and the 3C protease inhibition was defined as 100%×(A_HRP+drug+3C protease_-A_HRP++3C protease_)/(A_HRP+drug_-A_HRP+3C protease_). The dose that elicited an inhibition of 50% (IC_50_) was also calculated [37]. Rutin (0.5 mM), which has been proven to inhibit 3C protease, was used as a positive control.

### Statistics

All data are expressed as the means ± the SEs. The statistical significances of the differences in the mean values were assessed with *t*-tests following one-way analyses of variance (ANOVA). The asterisks indicate significance at the *P*<0.05 level.

## Results

### Cytotoxicity of luteoloside to RD cells

**Fig 1:**
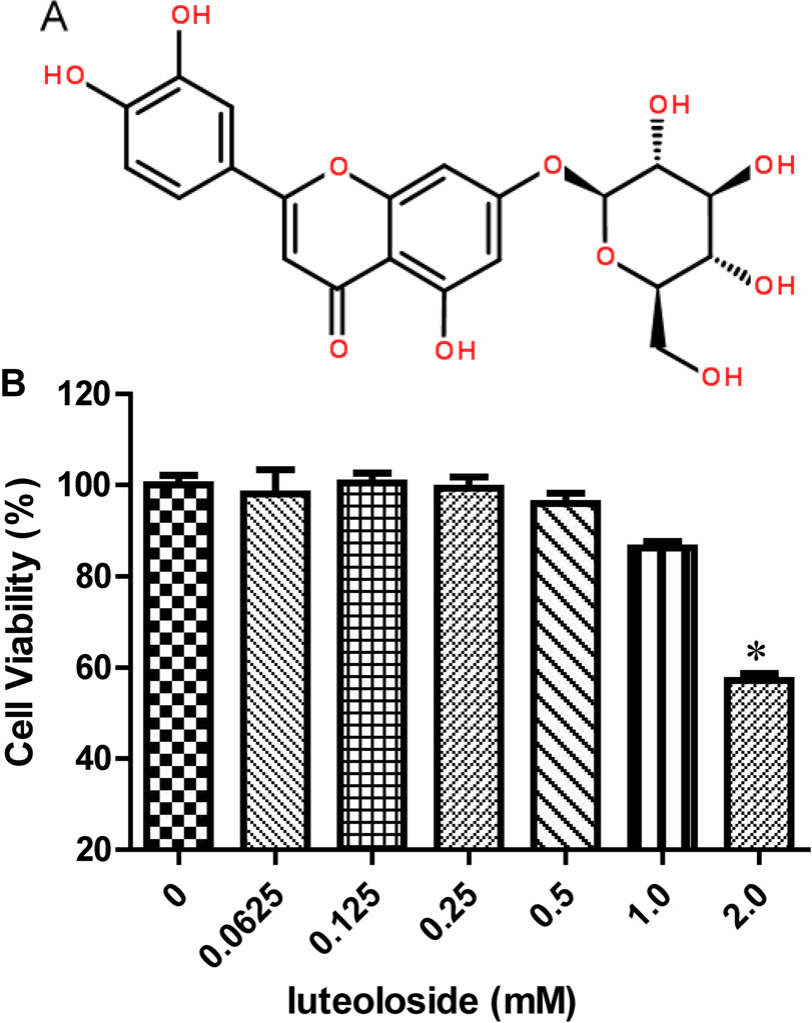
The molecular structure of luteoloside and its effects on RD cell viability. (A) The molecular structure of luteoloside. (B) Luteoloside was diluted as various concentrations as indicated in triplicate. The cytotoxicity of the compound was determined by MTS assay after drug-incubation for 48 h. The viability of cells upon DMEM medium without luteoloside (0 mM) was set as 100%. Data shown are the means ± SE from 6 independent measurements (*n* = 6). Asterisk meant the data differed from the blank (0 mM) significantly at *P*<0.05 level according to *t*-test.

The molecular structure of luteoloside is illustrated in Fig 1A. To clarify whether the inhibitory activity of luteoloside against EV71 was associated with its toxicity to the cells, the viabilities of RD cells were examined 48 h after luteoloside administration. Luteoloside exhibited no obvious toxicity to the cells at concentrations ≤1 mM (Fig 1B). Cell viability slowly decreased relative to the control as the concentration increased to 1.5 mM (Fig 1B, *P*<0.05). However, at ≥3 mM, luteoloside was significantly toxic to the RD cells (Fig 1B, *P*<0.01) with a CC_50_ of approximately 2.3 mM. Based on these results, we identified the nontoxic concentration range of luteoloside for RD cells.

### Luteoloside protects RD cells from EV71 infection

**Fig 2:**
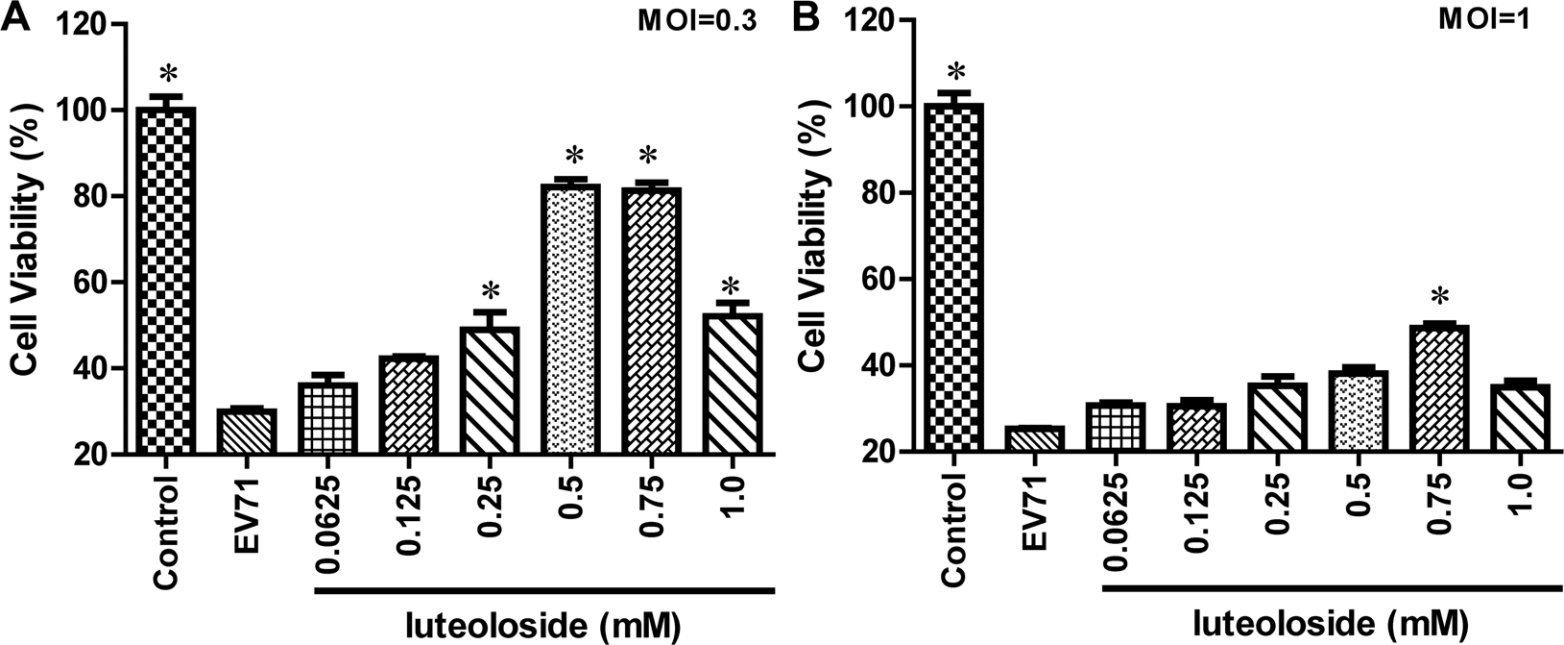
The antiviral effects of luteoloside against EV71 in RD cells. RD cells were infected 0.3 MOI (A) or 1.0 MOI (B) EV71 with or without different concentrations of luteoloside are shown. Uninfected cells were taken as control group. The cell viability was detected using MTS cell proliferation assay kit at 48 hpi. The viability of control group was set as 100%. Data shown are the means ± SE from 6 independent measurements (*n* = 6). Asterisk meant the data differed from the EV71 group significantly at *P*<0.05 level according to *t*-test.

The antiviral efficacy of luteoloside was evaluated using a RD cellular model. In a viability protection assay (Fig 2), luteoloside reduced the cytopathic effect (CPE) of EV71 infection in a dose-dependent manner. As illustrated in Fig 2A, 0.5 mM luteoloside elicited an inhibitory rate of 79.2% and significantly ameliorated the CPE induced by EV71 at the MOI of 0.3. At other doses, luteoloside also mitigated the CPE. For example, 0.75 mM luteoloside elicited effects on the EV71-induced CPE that were similar to those of the 0.5 mM concentration; specifically, the CPE was reduced by 73.3%. The results illustrated in Fig 2B indicate that 0.75 mM luteoloside rescued the cells from the CPE induced by EV71 at the MOI of 1.0 (*P*<0.05). However, the other concentrations of luteoloside elicit only slight mitigations of the CPE induced by the virus (Fig 2B). Taken together, these results indicate that 0.5 mM luteoloside appeared to be an effective dose against the administration of EV71 at the MOI of 0.3; thus, this dose was used in the subsequent experiments. Additionally, we observed an EC_50_ and selection index of approximately 0.43 mM and 5.3, respectively.

### The antiviral effects of luteoloside

**Fig 3:**
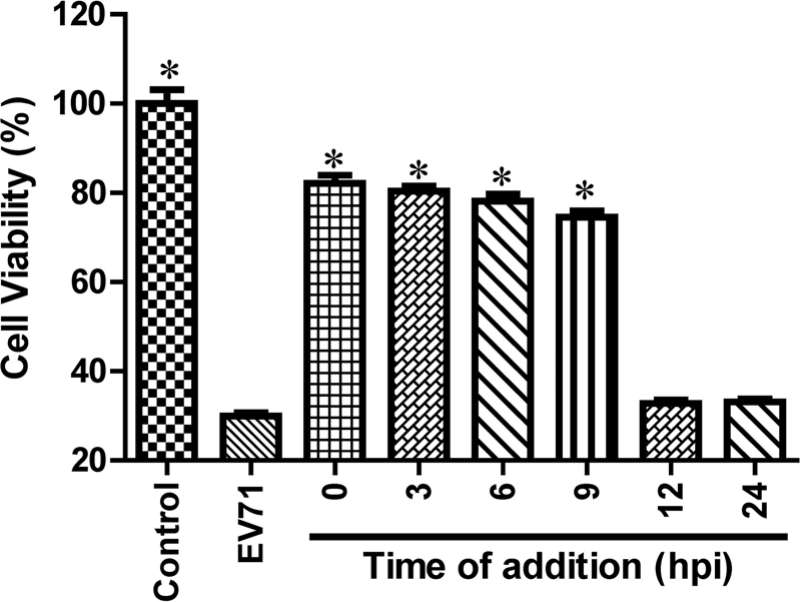
Luteoloside inhibited EV71 production in a time-dependent manner. RD cells were infected EV71 (MOI = 0.3). 0.5 mM luteoloside were added at the indicated time points, respectively. Uninfected cells were used as a control group. The cell viability was detected using MTS cell proliferation assay kit at 48 hpi. The viability of control group was set as 100%. Data shown are the means ± SE from 6 independent measurements (*n* = 6), asterisk means *P*<0.05 according to *t*-test comparing with EV71 group.

To investigate the antiviral effects of luteoloside, the compound was added to EV71-infected RD cells at various time points (0–24 hpi). As illustrated in Fig 3, the addition of luteoloside at 0–9 hpi markedly suppressed the EV71-induced CPEs (*P*<0.05). In contrast, when the compound was administered at 12–24 hpi, we observed limited inhibitory effects against EV71. Thus, the inhibitory effects of luteoloside against EV71 were only elicited following administration within the first 12 hours after infection, which suggests that luteoloside inhibits EV71 yield during the early stage of infection.

### Time -of-addition experiments and identification of the viral target of luteoloside

**Fig 4:**
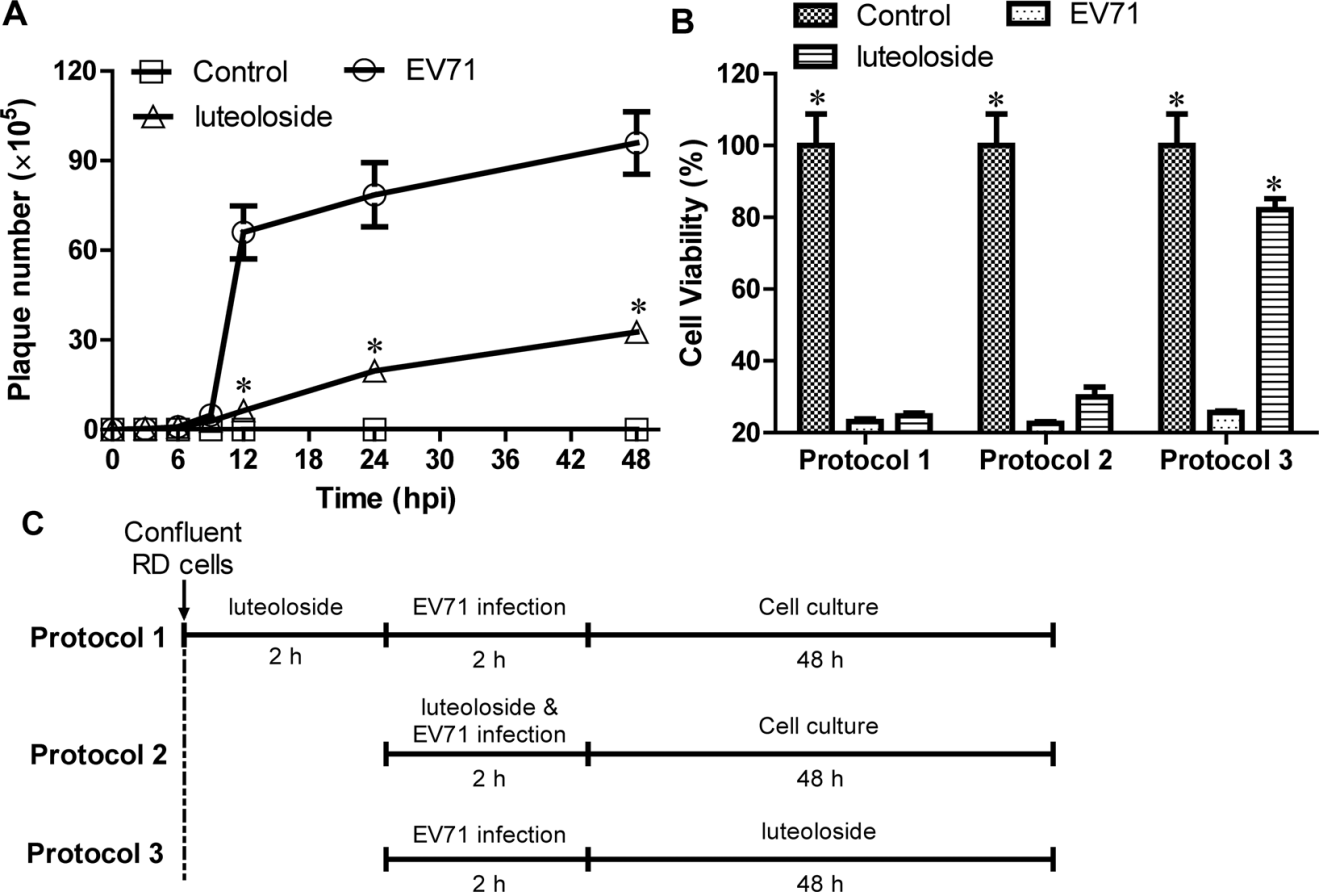
Luteoloside reduced EV71 production. (A) RD cells grown in 6-well-plate were infected with EV71 (MOI = 0.3) in the presence or absence of 0.5 mM luteoloside. The intercellular and extracellular virions were collected at the indicated time points for plaque reduction assay by freeze-thawing. (B) RD cells were infected with EV71 (MOI = 0.3). 0.5 mM luteoloside were supplemented to infected RD cells according to different protocols (C) respectively. The cell viability was detected using MTS cell proliferation assay kit at 48 hpi. The viability of control group was set as 100%. Data shown are the means ± SE from 6 independent measurements (*n* = 6). Asterisk meant the data differed from the EV71 group significantly at *P*<0.05 level according to *t*-test.

To confirm the inhibitory effects of luteoloside on EV71 production and more precisely determine the stage of viral infection during which the compound is effective, we performed a time course assay. Intercellular and extracellular viral particles were collected at 0–48 hpi for use in plaque forming assays. As illustrated in Fig 4A, the EV71 titre slowly increased from 0–9 hpi. From 9–12 hpi, we observed a local peak in the viral titre that was followed by a gradual increase, which indicated the rapid propagation of EV71 during these hours. However, the addition of 0.5 mM luteoloside significantly blocked the peak growth at 9–12 hpi (Fig 4A, *P*<0.05). The addition of luteoloside prior to or simultaneously with viral inoculation (2 h incubation; Protocols 1 and 2, respectively, Fig 4C) failed to protect the cells from EV71 infection (Fig 4B). The administration of luteoloside after 2 h of incubation with EV71 (Protocol 3, Fig 4C) prevented the virus-induced CPE (*P*<0.05, Fig 4B). These results demonstrated that luteoloside acted by effectively suppressing EV71 production in RD cells rather than by stimulating cellular defences or antagonizing viral absorption.

### Luteoloside suppresses *VP1* gene and protein synthesis

**Fig 5:**
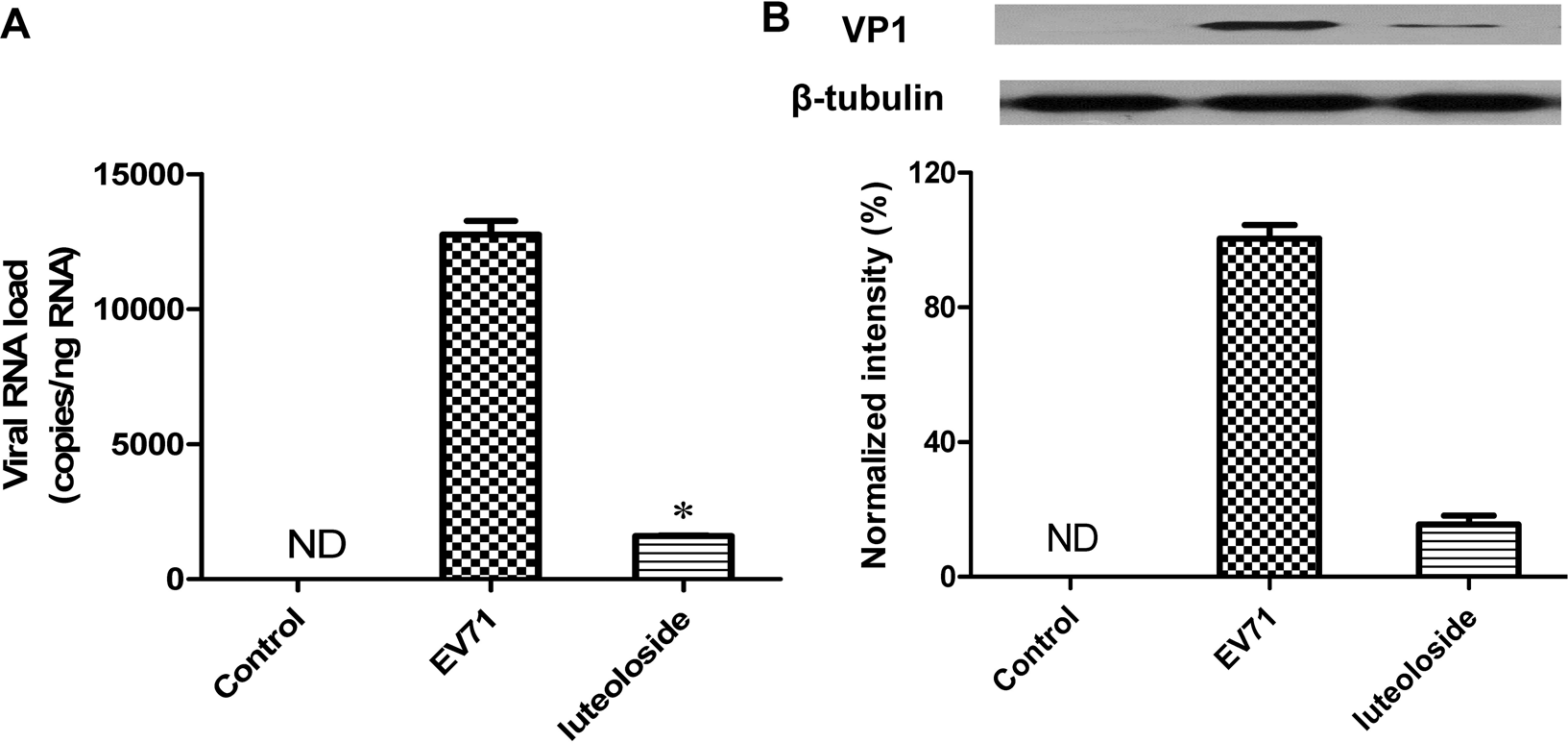
Luteoloside inhibited the production of viral proteins and nuclear acids. RD cells grown in 6-well-plate were infected with EV71 (MOI = 0.3) in the presence or absence of 0.5 mM luteoloside. Cells were lysed for total RNA and protein extraction at 12 hpi, respectively. (A) The RNA load was determined using the real-time PCR kit specific to *VP1* gene. Data shown are the means ± SE from 6 independent measurements (*n* = 6). Asterisk meant the data differed from the EV71 group significantly at *P*<0.05 level according to *t*-test. (B) Protein samples, unified to 30 μg, were subjected to 12.5% SDS-PAGE and then transferred to PVDF membrane to detect the level of EV71 VP1 protein. The amount of β-tubulin was used as internal standard. VP1 band intensity was analyzed and normalized to corresponding band intensity of β-tubulin. The band intensity of EV71 group was set as 100%. Data shown are the means ± SE from 3 independent measurements (*n* = 3).

To further investigate the inhibitory effects of luteoloside on EV71 yield, *VP1* mRNA transcript and protein levels were assessed. As illustrated in Fig 5A, luteoloside suppressed EV71-induced *VP1* mRNA transcripts in RD cells by approximately 8-fold at 12 hpi, and this effect was similar to the reduction observed at this time point in our plaque assay. To measure the VP1 protein levels, we utilized western blot analyses. Our results revealed that VP1 protein expression increased during EV71 infection at 12 hpi. However, the addition of luteoloside reduced the protein expression by approximately 6-fold (Fig 5B). Thus, these data indicate that luteoloside suppressed transcription, which subsequently reduced protein translation during EV71 infection. However, direct effects of luteoloside on protein translation could not easily be ruled out. Therefore, our results demonstrated that the administration of luteoloside inhibited the production of EV71 in RD cells.

### Luteoloside inhibits the EV71 3C protease *in vitro*

**Fig 6:**
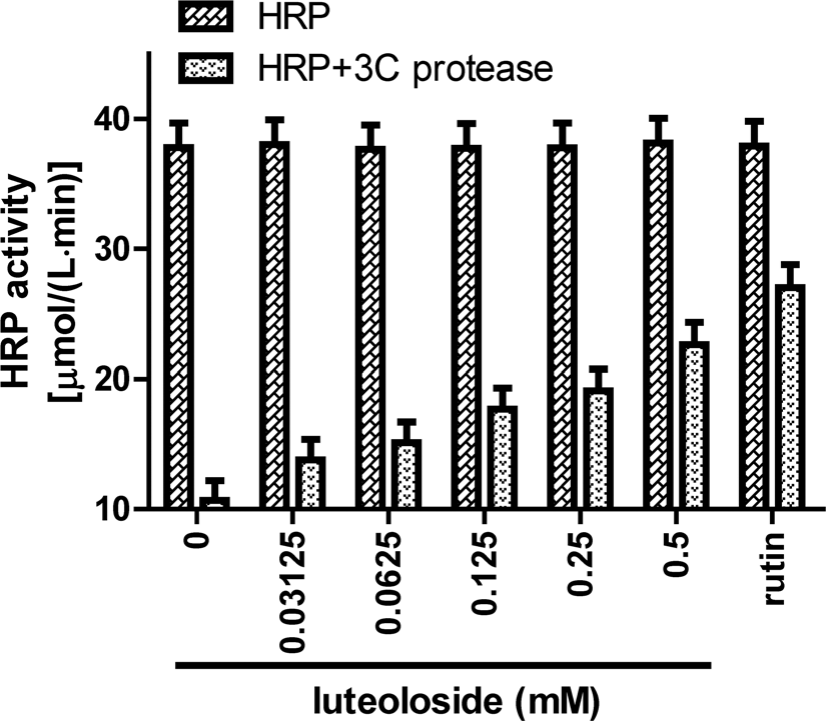
Dose-dependent inhibitory of luteoloside on EV71 3C protease activity *in vitro*. Two-fold dilutions of luteoloside were added in 3C protease analysis system. The enzymatic activity of HRP exposed to various treatments was calculated. Blank (0 mM luteoloside) and 0.5 mM rutin were set as negative and positive control, respectively. Data shown are the means ± SE values of 3 independent experiments (*n* = 3).

To identify the potential antiviral mechanism(s) and target(s) of luteoloside, the effects of the compound (0–0.5 mM) on 3C protease activity were investigated. For this enzymatic assay, we used an HRP type VI-A containing a 3C protease cleavage site (Gln-Gly) as the 3C protease substrate. As illustrated in Fig 6, HRP activity decreased in the presence of 3C protease, and this effect was attributed to the enzymatic cleavage of HRP by the protease. The suppression of HRP activity by 3C protease was dose-dependently relieved by the addition of luteoloside. These results were similar to the observed effects of rutin (IC_50_≈0.4 mM), which is a well-known 3C protease inhibitor [32]. All of the tested luteoloside concentrations failed to inhibit HRP activity. Taken together, these data indicate that luteoloside is a 3C protease inhibitor. Additionally, the IC_50_ of luteoloside was 0.36 mM. Based on the results of our HRP-based enzymatic assay, luteoloside appears to inhibit the 3C protease in a dose-dependent manner *in vitro*.

## Discussion

*Lonicera japonica* is a well-known traditional Chinese herb that has been widely used in HFMD treatments [38] due to its antiviral activity against HFMD pathogens, including coxsackievirus [39]. Luteoloside is a compound that can be extracted from the herb and has previously been reported to exhibit antiviral activities against the influenza virus [1,2]. However, the evidence for its effects on enteroviruses, such as EV71, is still limited. The data from the present study indicate that luteoloside blocks 3C protease activity and subsequently inhibits EV71 production *in vitro*.

RD cells have previously been demonstrated to be sensitive to EV71 infection [40], and EV71 has been reported to induce autophagy and apoptosis in RD cells following infection [34,41]. Therefore, in the present study, we selected RD cells as a model to better understanding the effects of luteoloside on EV71 [42,43]. First, we investigated the cytotoxicity of luteoloside on RD cells and identified the non-toxic dose range as ≤1 mM. Therefore, in the subsequent further experiments, we used a luteoloside dose that was non-toxic to RD cells. According to previous reports, EV71 infections cause CPE in RD cells [33,44]. In the present study, we demonstrated that the administration of luteoloside dose-dependently prevented EV71-induced CPE. The inhibitory effects of this compound on the replication of the influenza virus have also been demonstrated [2], which suggests that luteoloside exhibits a broad spectrum of antiviral activities. Furthermore, luteolin, which lacks the glucoside moiety found in luteoloside, has also been demonstrated to inhibit EV71 production *in vitro* [45]. Collectively, these data indicate that luteoloside exhibits antiviral activity against EV71.

Time course analysis indicated that the addition of luteoloside at 0–9 hpi significantly inhibited the EV71-induced CPE, which contrasts with the minimally inhibitory effect observed following the addition of luteoloside at 12–24 hpi (Fig 3). Previous studies have reported the viral kinetics of EV71 in RD cells [46]. Thus, the inhibitory effects of luteoloside on EV71 production in the early stage of infection (<12 hpi) could be determined. Furthermore, the peak of the EV71 viral titres was suppressed by the administration of luteoloside (Fig 4A), which explains the inhibition of viral viability by luteoloside and the subsequent prevention of CPE (Fig 3). Interestingly, the addition of luteoloside protected the cells from EV71 infection only after viral absorption (Fig 4B) and enabled luteoloside to effectively inhibit the EV71 yield. A similar result has been observed with chlorogenic acid, which is an EV71 inhibitor [31]. The addition of luteoloside was only able to reduce the virus-induced CPE after infection, which indicated that luteoloside inhibits EV71 production in RD cells at the stages of and subsequent to nuclear acid transcription and protein translation. In contrast, the potential effects of the compound on viral attachment, absorption, and penetration could not be deduced in the present study. Therefore, we conclude that the EV71 yield was reduced in the presence of luteoloside. Moreover, we investigated the inhibitory effects of luteoloside on *VP1* mRNA and protein syntheses. The VP1 protein contains the majority of the EV71 epitopes and is used to detect EV71 infections [22,47]. In the present study, we observed marked reductions in the productions of mRNA transcripts and proteins following the addition of luteoloside. Similarly, the down-regulation of VP1 expression has also been observed following the addition of the known EV71 inhibitor lycorine [47].

The 3C protease plays a major role in the EV71 life cycle; it cleaves the precursor polyprotein into structural and functional proteins [17] and activates the 3D protein that is responsible for RNA replication [18]. A previous study [32] developed an enzymatic assay for 3C protease detection using an HRP-type VI-A (containing a 3C protease cleavage site) as the C3 protease substrate. Rutin is a member of the flavonoid family that also exhibits antiviral activities [32,48,49]. The molecular effects (Figure A in S1 File) and cytotoxicity (Figure B in S1 File, CC_50_ = 1.19 mM) of rutin on RD cells are illustrated. Rutin inhibits EV71 replication by blocking 3C protease activity (IC_50_≈0.4 mM) [32]. The results of the present study indicate that luteoloside dose-dependently inhibits 3C protease activity in a manner similar to that of rutin. Additionally, no inhibitions of HRP activity by luteoloside or rutin were observed. The IC_50_ of luteoloside was 0.36 mM, which is approximately equal to the EC_50_ calculated based on the *in vitro* RD cell assay; these findings further support the antiviral effects of luteoloside. Several antiviral compounds have been reported to act as 3C protease inhibitors and antagonists [50,51]. The antiviral activity of luteoloside against the influenza virus has also been previously reported [2]. The abilities of this compound to inhibit EV71 via other mechanisms should be further evaluated in future studies. Taken together, our results indicate that luteoloside inhibits EV71 yield by blocking 3C protease activity.

In summary, this study indicated that luteoloside has antiviral effects against EV71 that are mediated by blocking 3C protease activity, and these effects subsequently inhibited EV71 production. These results suggest that luteoloside may be a moderately potent agent that could be utilized for the treatment of EV71 infection.

## Supporting Information

S1 File The molecular structure of rutin and its effects on RD cells viability. (Figure A in S1 File) The molecular structure of rutin. (Figure B in S1 File) Rutin was diluted as various concentrations as indicated in triplicate. The cytotoxicity of the compound was determined by MTS assay after drug-incubation for 48 h. The viability of cells upon DMEM medium without luteoloside (0 mM) was set as 100%. Data shown are the means ± SE from 3 independent measurements (*n* = 3). Asterisk meant the data differed from the blank (0 mM) significantly at *P*<0.05 level according to *t*-test.
(DOC)
