# Supplementary material for: Luteoloside Acts as 3C Protease Inhibitor of Enterovirus 71 In Vitro
Source: PLoS One. 2016 Feb 12;11(2):e0148693. doi: 10.1371/journal.pone.0148693 (PMC4752227; doi:10.1371/journal.pone.0148693)
Supplement: S1 File — (Figure A in S1 File) The molecular structure of rutin. (Figure B in S1 File) Rutin was diluted as various concentrations as indicated in triplicate. The cytotoxicity of the compound was determined by MTS assay after drug-incubation for 48 h. The viability of cells upon DMEM medium without luteoloside (0 mM) was set as 100%. Data shown are the means ± SE from 3 independent measurements (n = 3). Asterisk meant the data differed from the blank (0 mM) significantly at P<0.05 level according to t-test. (DOC) [file pone.0148693.s001.doc]

Luteoloside acts as 3C Protease Inhibitor of Enterovirus 71 *In Vitro*

Zeyu Cao, Yue Ding, Zhipeng Ke, Liang Cao, Na Li, Gang Ding, Zhenzhong Wang, Wei Xiao*

State Key Laboratory of New-tech for Chinese Medicine Pharmaceutical Process, Jiangsu Kanion Pharmaceutical Co., Ltd., Lianyungang, Jiangsu, China

**Supporting Information**

**S1 Figure. The molecular structure of rutin and its effects on RD cells viability**

**S1 Figure. The molecular structure of rutin and its effects on RD cells viability.** (A) The molecular structure of rutin. (B) Rutin was diluted as various concentrations as indicated in triplicate. The cytotoxicity of the compound was determined by MTS assay after drug-incubation for 48 h. The viability of cells upon DMEM medium without luteoloside (0 mM) was set as 100%. Data shown are the means ± SE from 3 independent measurements (*n*=3). Asterisk meant the data differed from the blank (0 mM) significantly at *P*<0.05 level according to *t*-test.
